# The Role of Sugars in Plant Responses to Stress and Their Regulatory Function during Development

**DOI:** 10.3390/ijms23095161

**Published:** 2022-05-05

**Authors:** Philippe Jeandet, Magda Formela-Luboińska, Mateusz Labudda, Iwona Morkunas

**Affiliations:** 1 Research Unit “Induced Resistance and Plant Bioprotection”, Department of Biology and Biochemistry, Faculty of Sciences, University of Reims, EA 4707–USC INRAe 1488, SFR Condorcet FR CNRS 3417, P.O. Box 1039, CEDEX 02, 51687 Reims, France; 2 Department of Plant Physiology, Poznań University of Life Sciences, Wołynska 35, 60-637 Poznań, Poland; magda.formela-luboinska@up.poznan.pl; 3 Department of Biochemistry and Microbiology, Institute of Biology, Warsaw University of Life Sciences-SGGW, Nowoursynowska 159, 02-776 Warsaw, Poland; mateusz_labudda@sggw.edu.pl

Due to their role as energy and carbon sources and their regulatory functions, sugars influence all phases of the plant life cycle, interact with other signaling molecules, including phytohormones, and control plant growth and development [1,2,3]. The levels of sugars in plant cells, their transport, utilization, and storage are precisely regulated and strongly dependent on cell physiological activity, plant organs, environmental conditions, circadian rhythms, and plant developmental stages [4]. The perception of the signals induced by sugars can take place in the apoplast and during transport across membranes or inside the cell, e.g., in the cytosol, and may involve glucose and sucrose membrane transporters, invertases, and hexokinases (HXK) as conserved glucose sensors, and changes in the AMP to ATP ratio [5,6,7]. Rolland et al. [1] reported that sugar signals activate multiple HXK-dependent and HXK-independent pathways and provided evidence of the effects of sugar signals on transcription, translation, protein stability, and enzymatic activity. In turn, Snf1-related kinases (SnRKs), calcium-dependent protein kinases (CDPKs), the mitogen-activated protein kinase *(*MAPK*) *cascade**, specific protein phosphatases (PPs), phytohormones, and calcium ions are involved in sugar-induced signal transduction [1]. The plant’s ability to monitor and respond to sugar cellular levels may act as a controlling mechanism, integrating the influence of environmental conditions with internal developmental programs directly regulated by phytohormones [8,9].

Many environmental stimuli, the intensity of which may vary according to climate change, may influence various biochemical processes, frequently interfering with the balanced partitioning of sugars within plant cells and their transport from source organs to sink organs. The main currency in sugar exchanges in higher plants is represented by sucrose, the major product of photosynthesis in the leaves, which is transported throughout the plant, which involves sucrose uptake transporters (SUT/SUC: active sucrose/H^+^ symporters) and export transporters SWEETs (hexose and sucrose transporters) [10,11]. Sucrose can be degraded by several enzymes (invertases and sucrose synthase) or resynthesized from its degradation products in the so-called “sucrose cycle” [12].

Numerous studies have also shown that sugars play a key role in plant defense responses to various abiotic and biotic stress factors [13,14]. It is well documented that sugars are not only the main substrates utilized in respiration processes supplying energy for cellular defense responses against pathogens, but also provide the carbon skeleton for the synthesis of defense compounds including secondary metabolites such as flavonoids, stilbenes, and lignins [15,16]. In addition, saccharides such as sucrose, glucose, fructose, and trehalose represent metabolic signaling molecules in host plant cells, which induce the expression of many genes, namely defense genes [17,18,19].

A high level of sugars in plant tissues enhances the plant immune response against fungal pathogens. Sugars probably function as priming molecules, leading to pathogen-associated molecular pattern (PAMP)-triggered immunity (PTI) and effector-triggered immunity (ETI) in plants [20]. It has been demonstrated that the presence of sucrose and monosaccharides enables plants to stimulate efficient defense mechanisms against fungal pathogens. This is consistent with the novel concepts of “sweet immunity” and “sweet priming” [21,22], postulating that saccharides play a key role as priming agents, inducing the resistance of higher plants to both biotic and abiotic stresses. Recent progress made by Van den Ende’s group in sugar research has also provided important evidence regarding the contribution of fructans in the adaptation of plants to abiotic stress and in plant immune responses to pathogens (see this Special Issue). The papers presented hereafter in this Special Issue thus illustrate the central role of sugars in plant development and in the improvement of crop yields as well as in the defense responses to stresses and the impact of environmental and climatic conditions on the relationship between sugar metabolism and yielding. Other papers regarding the involvement of sugars as signaling molecules in the processes regulating growth and development are also presented.

Sucrose is one of the main products of photosynthesis in leaves. Once synthesized, it is transported through the phloem from leaves to all parts of the plant. Sucrose not only plays the role of a carbon carrier between source and sink tissues, but also acts as a long-distance signal for the control of many processes involved in plant development such as apical dominance and root growth. It also intervenes in the production/consumption balance of plant carbohydrates. In their review article, Aluko et al. [23] discussed the utilization of sucrose for crop yield improvement, analyzed how alterations of the source to sink sugar balance may impede physiological and developmental processes, and explored factors that control photoassimilate partitioning within the entire plant, that is, translocation mechanisms and the utilization of sucrose, at the sink organs. The influence of various environmental parameters on sugar transport (CO_2_, light, temperature, drought, and nutrient availability) was also discussed. Sugar transporters (SUTs and SWEETs) and sucrose are involved in mitigating environmental stresses such as elevated CO_2_, cold, and drought. A significant part of this article also described the strategies dedicated to increasing the efficiency of photosynthetic assimilation and integrated approaches to crop-yield improvement.

The adjustment of plant growth and development goes through sophisticated regulatory processes involving intricate crosstalk between sugars and hormones. In their paper, Wang et al. [24] described the complex convergent and divergent inter-relationships that take place between cytokinin signaling and sugars in many aspects of the plant life cycle, including seed development and germination, leaf senescence, root and shoot branching, as well as flowering. The interplay between sugars and cytokinins may lead to antagonistic or synergistic interactions depending on the morphogenetic and the developmental phases of the plant. Synergistic effects were described in flowering, shoot branching, and functioning of the shoot meristem though antagonistic interactions occurred between sugars and cytokinins in the functioning of the root meristem. The complexity of the crosstalk between sugars and cytokinins in controlling plant development and morphogenesis was thus analyzed in this paper.

Grape berries can accumulate exceptionally high sugar contents, rising from 10 g/kg of fresh weight at the beginning of the maturation time to hundreds of grams per kilogram at full maturity. In their review article, Walker et al. [25] paid attention to both sucrose metabolism and transport in grapevines. The role of various enzymes involved in sucrose breakdown and sucrose resynthesis such as sucrose synthase, neutral and acid invertases within the so-called “sucrose cycle”, sucrose translocation within and between organs, as well as its associated metabolism in grape pericarp during ripening, were described thoroughly in the light of a cross-species comparison including tomato, potato, carrot, maize, and apple.

The accumulation, metabolism, and transport of sugars, and especially those of sucrose, are strongly dependent on both environmental and physical parameters such as temperature, CO_2_, photoperiod, and the circadian rhythm as well as interactions with plant hormones. Four articles discussed the implication of cold, photoperiod, and hormonal control in soluble sugar and sucrose contents in relation to plant development phases [26,27,28,29].

Acclimation to cold exposure requires changes in carbohydrate metabolism such as the accumulation of soluble sugars and starch hydrolysis in chloroplasts. The work of Orzechowski et al. [26] discussed the very early response of leaves of potato plants exposed to cold stress (2 °C for 12 h), decoupling the effects of light and temperature. The expression of enzymes involved in starch degradation, starch-related dikinases (StGND and StPWD) acting on starch turnover, as well as glucan phosphorylase, amylase, invertase, and disproportioning enzyme 2 activities were increased following adaptation to cold exposure. A modification of the carbohydrate metabolism in leaves leading to an augmentation of soluble sugar accumulation was also demonstrated.

Morphological and developmental features in plants imply complex sugar regulation processes. Li et al. [27] showed that the exogenous application of the synthetic plant cytokinin 6-benzyladenine but not the natural gibberellin GA3, and a long-day photoperiod, but not temperature, induce runner formation in an octaploid cultivated strawberry variety from South Korea. This asexual propagation trait was mediated through the upregulation of the soluble sugar content. A proteomic analysis also revealed that a total of 16 proteins were differentially expressed in the runner-activated strawberry plants versus plants without runners, of which, the major protein group related to carbohydrate metabolism and photosynthesis (sucrose synthase 2 and glucan endo-1,3-β-glucosidase). This study thus clearly evidenced the positive correlation that exists between soluble sugar content and runner induction in strawberry.

Micro RNA172 (miR172) as well as micro RNA156 (miR156) are involved in the regulation of the transitions that occur between different developmental stages in plants. Garg et al. [28] demonstrated that tomato plants silenced in the sucrose transporter gene *StSUT4* display higher levels of miR172 as compared to the wild type and display similar phenotypical traits regarding flowering induction and tuberization as miR172-overexpressing plants. Moreover, the contents of miR172 were increased by high levels of sucrose, suggesting the sucrose-inducible character of miR172 expression. A strong interlink was described between the sugar status of potato plants and the downstream pathways regulating flowering induction and tuberization, including miR172 and StSUT4.

Improving grain yield represents a challenge in wheat production. Thus, addressing the diurnal patterns of sugar transport in wheat during the period of grain filling is of major concern. Al-Sheikh Ahmed et al. [29] investigated the possible diurnal changes in water-soluble carbohydrates that occur in correlation with different expression levels of the sucrose transporter *TaSUT1* gene in two different wheat varieties, Kauz and Westonia. It was suggested that a higher expression of *TaSUT1* in leaves that correlates well with the high sucrose levels observed in the variety Kauz can contribute to a grain weight increase in this variety.

The first identification of the sucrose transporter family from pomegranate (*Punica granatum* L.) was reported in the work of Poudel et al. [30]. From a phylogenetic tree analysis, pomegranate *SUT* genes were found to be divided into three major groups varying in the number of exons and introns. The promoter regions of these genes were characterized by their richness in MYB cis-elements. Finally, a functional analysis of the *SUT* gene *PgL0145810_-_1* revealed its involvement in the regulation of the seed developmental process, especially the seed hardness trait in pomegranate, likely playing a role in lignin synthesis.

In addition to their fundamental roles as donors of carbon skeletons and substrates for respiration processes, sugars are also implicated in intricated plant network responses to biotic and abiotic stresses. Briefly, sugars intervene in the regulation of signaling molecules related with plant defenses, plant immunity, the phenomena of priming, and sweet immunity. Several papers published in this Special Issue are dedicated to these aspects [31,32,33,34]. The Morkunas group published two successive works on the role of sugars both in the mechanisms of pathogenicity of *Fusarium oxysporum* f. sp. *lupini* to yellow lupine and in the regulation of the levels of endogenous signaling molecules during the defense responses of that plant to *F. oxysporum*. In a first approach, Formela-Luboińska et al. [31] showed that the production of ergosterol, a membrane fungal sterol taken here as a fungal indicator of the mycelial growth of *F. oxysporum* as well as levels of the mycotoxin moniliformin, were decreased in embryo axes of yellow lupine infected with this fungus and cultured in media under a sugar deficit. In addition, soluble sugars were found to provoke an inhibition of the sporulation of this pathogen, showing they can intervene in limiting the development and spread of systemic pathogens such as *F. oxysporum*. The interplay of sugars in the mechanisms of pathogenicity on yellow lupine, such as a possible catabolic repression by sugars of fungal polygalacturonase gene expression, was also discussed.

In a second article, Formela-Luboińska et al. [32] emphasized the role of sucrose and various monosaccharides as signaling compounds for the regulation of phytohormones, abscisic acid (ABA), ethylene, salicylic acid (SA), and its glucoside (SAG), as well as phenylalanine ammonia lyase (PAL), benzoic acid 2-hydroxylase (BA2H), and superoxide dismutase (SOD) activities in embryo axes of yellow lupine challenged with the hemibiotrophic fungus, *F. oxysporum*. Positive correlations were observed between the sugar levels and the post-infectious production of SA, SAG, ABA, and ethylene, as well as the increased activity of PAL, BA2H, and SOD, with all of these parameters participating in the defense responses of yellow lupine to *F. oxysporum*.

Sugars contribute to plant immunity, with the so-called “sweet immunity” concept inferring that sugar metabolism and signaling are capable of boosting the defense responses of plants. Exogenous applications of structural carbohydrates were shown to improve plants’ resistance to pathogens, directly or through the stimulation of the defense mechanisms of plants themselves. The concept of sweet immunity was established via the effect of exogenous fructans regarding apple (*Malus* x *domestica* Borkh.) susceptibility to apple scab (*Venturia inaequalis*) in the work of Svara et al. [33]. The exogenous application of specific fructans and levans on apple leaves was shown to be able to reduce the development of this pathogen through a significant decrease in its sporulation. In addition, the direct inhibition of the fungal growth was observed in vitro on peptone dextrose agar plates supplemented with fructans. Levans thus boost the resistance of apple leaves to apple scab, although the mechanisms involved in this resistance remain unelucidated. A possible application of this technique for use in apple scab management in orchards was also discussed.

Chitinases are known as crucial components of plant innate immunity. A *CaChiIII7* chitinase gene, which takes part in the regulation of the hypersensitive response and defense of pepper (*Capsicum annulum* L.) to anthracnose (*Colletotrichum acutatum*) infection, was newly identified in the article of Ali et al. [34]. This gene, which possesses repeated chitin-binding domains, encodes a chitinase transcriptionally stimulated upon infection by *C. acutatum*. Moreover, this *CaChiIII7* putative chitinase gene was shown to share high sequence homology with other chitinase families. The functional characterization of this chitinase was achieved through knockdown experiments of the *CaChiIII7* gene in pepper plants, resulting in their increased susceptibility to *C. acutatum* and demonstrating the involvement of this new chitinase in pepper responses to anthracnose.

All of these articles thus illustrate the central role played by sugars in the physiology, the development, and the defense responses of plants.

## Data Availability

Not applicable.
